# Parkinsonism and Dystonia Are Prevalent and Concomitant Movement Disorders in a Cohort of Patients with Rett Syndrome

**DOI:** 10.1002/mdc3.70158

**Published:** 2025-05-30

**Authors:** Silvia Boeri, Giulia Prato, Maria Grazia Calevo, Silvia Casabona, Lino Nobili, Elisa De Grandis

**Affiliations:** ^1^ Department of Neuroscience, Rehabilitation, Ophthalmology, Genetics, Maternal and Child Health (DINOGMI), University of Genoa Genoa Italy; ^2^ Child Neuropsychiatry Unit IRCCS Istituto Giannina Gaslini Genoa Italy; ^3^ Epidemiology and Biostatistics Unit Scientific Direction, IRCCS Istituto Giannina Gaslini Genoa Italy

**Keywords:** dystonia, *MECP2*, movement disorders, parkinsonism, Rett syndrome

## Abstract

**Background:**

Rett syndrome (RTT) is a rare neurodevelopmental disorder linked with *MECP2* variants, frequently presenting with movement disorders (MDs).

**Objectives:**

This study examined the frequency, types, and associations of MDs with RTT characteristics and severity.

**Methods:**

Twenty female patients (median age 11 years, range 3–40) with *MECP2* variants were recruited and assessed using disease severity and MD scales, alongside videotaped neurological examinations.

**Results:**

Prevalent MD was hypokinetic in 55% of the patients and hyperkinetic in 45%. Parkinsonism and dystonia were the most common, coexisting in 75% of patients. Higher Movement Disorders‐Childhood Rating Scale (MD‐CRS) scores correlated with greater severity on the Rett Assessment Rating Scale (RARS) (*P* = 0.04). Dystonia was significantly associated with respiratory (*P* < 0.05) and gastrointestinal problems (*P* = 0.02).

**Conclusions:**

The study identified parkinsonism and dystonia as the prevalent MDs in RTT, emphasizing the association between dystonia and gastrointestinal/respiratory problems and reflecting the complex nature of MDs in RTT.

Rett syndrome (RTT, OMIM #312750) is a rare neurodevelopmental disorder that predominantly affects females, with a prevalence of 5–10 per 100,000.^1^ RTT requires specific clinical criteria (redefined by Neul et al), although genetic testing may confirm the diagnosis.^2^, ^3^

Genetically, RTT is linked to *MECP2* variants in 95% of typical cases. Mutations in *CDKL5* and *FOXG1* account for atypical forms.^4^, ^5^, ^6^

Rett progression is classified into four stages (early stage, developmental regression, pseudostationary period, and late motor deterioration).^7^, ^8^ RTT symptoms include epilepsy and gastrointestinal, cardiovascular, breathing, sleep, and movement disorders (MDs), in addition to hand stereotypies, the hallmark of the syndrome.^2^, ^9^, ^10^

Movement disorders (MD) in RTT evolve with disease stage and patient age, encompassing hypokinetic (eg, Parkinson‐like rigidity) and hyperkinetic (eg, dystonia, tremor) forms, primarily affecting gait.^11^ Singh et al's meta‐analysis analyzed MD progression,^12^ Brunetti and Lumsden reported dystonia and gait abnormalities as the most frequent MDs in RTT.^11^

Understanding the characteristics of MDs in RTT and their correlation with other aspects of the disease is of clinical relevance, potentially improving patient quality of life through targeted interventions and therapies.

This study aimed to analyze the frequency and types of MD in RTT with *MECP2* variants and evaluate their associations with disease severity and other symptoms of RTT.

## Methods

In this single‐center retrospective observational study conducted between January 2022 and June 2023, patients were selected from the IRCCS Gaslini Children's Hospital database, which counted 97 patients. Female patients over 3 years of age carrying pathogenic *MECP2* variants were included. Atypical RTT without *MECP2* variants or patients carrying mutations in CDKL5 and FOXG1 genes were excluded.

To assess clinical severity and MD in patients with RTT, we used:

- Rett Assessment Rating Scale (RARS): severity levels are identified as mild (0–54), moderate (55–80), and high (81–128).^13^
- Clinical Severity Scale (CSS): scores above 21 indicated greater RTT severity.^14^
- Hand Apraxia Scale: scores ranging from 5 to 10 indicated a better manual function.^15^
- Movement Disorders‐Childhood Rating Scale (MD‐CRS): distinguished prevalent and additional MDs, and included a general assessment ‐Part I‐ (scores >30 indicated severe MD) and an MD assessment ‐Part II‐ (scores >14 indicated greater severity).^16^
- Burke‐Fahn‐Marsden Dystonia Rating Scale (BFMDRS): distinguished dystonia types and frequency, with severity graded as mild (0–40), moderate (41–80), and severe (81–120).^17^
- International Cooperative Ataxia Rating Scale (ICARS): consisting of five subscales. We administered the posture and gait subscale (scores between 18 and 34 indicated severe ataxia).^18^

Neurological examinations were video‐recorded. A child neuropsychiatrist evaluated each patient, performing neurological and motor assessments and administering movement and clinical rating scales. Two independent neuropsychiatrists (one specialist in MDs and one experienced in RTT) reanalyzed the videos, filling the scales. Discrepancies were resolved by averaging the three evaluators’ scores. Based on neurological and motor examination and administration of the MD‐CRS (Part I), we observed a prevalent (or primary) and an additional MD in patients, the latter being characterized by lower intensity and/or frequency. We identified MDs of hypokinetic or hyperkinetic type, often coexisting in patients. Patients with hypokinetic MDs exhibited bradykinesia, rigidity, hypomimia, postural instability, and freezing episodes (in ambulatory patients). These are typical features of Parkinson's disease, according to the clinical diagnostic criteria of the International Parkinson and Movement Disorder Society,^19^ therefore, having observed such clinical characteristics in our pediatric patients led us to use secondary parkinsonism in line with the most recent evidence supporting its use in the pediatric population.^20^

Among the hyperkinetic MDs, we identified dystonia, chorea, and tremor. We classified dystonia according to severity, location, and temporal pattern, based on the Albanese et al consensus^21^ and BFMDRS scores.

For each patient, the presence of features commonly associated with the syndrome (epilepsy, gastrointestinal, respiratory, sleep disorders, etc.) was assessed based on parental reports, medical records, assessments performed by medical specialists, and instrumental examinations (eg, polysomnography, EEG).

The study was conducted in accordance with the Declaration of Helsinki of 1964, and the parents or legal guardians of the participants provided signed video informed consent.

Descriptive statistics were generated for the whole cohort. Data are expressed as means and standard deviations for continuous variables and as absolute or relative frequencies for categorical variables. The distribution of the data was analyzed using the Kolmogorov–Smirnov test. Non‐parametric statistics were considered appropriate. Differences between groups were evaluated using the Mann–Whitney U test for continuous variables. Spearman correlation was calculated to assess the correlation between RARS scale and MD scales. A P value of <0.05 was considered to indicate statistical significance; all P values were based on two‐tailed tests. Statistical analysis was performed using the Social Sciences Statistical Package for Windows version 20 (SPSS Inc. Chicago, IL).

## Results

Twenty female patients with a median age at the time of evaluation of 11 years (range 3–40 years) were recruited. All patients had pathogenic variants in the *MECP2* gene. The patients were divided according to the stage of the disease: five were in stage II, twelve in stage III, and three in IV (no patients were in stage I).

Administration of the clinical severity scales showed that, according to RARS, 50% (10/20) of patients had moderate disease and 50% (10/20) had severe disease; 85% (17/20) of subjects had high CSS scores (Tables S1 and S2). The hand apraxia scale revealed that 75% (15/20) of the participants had a missing or extremely reduced hand function, and 95% (19/20) had hand stereotypies, pathognomonic feature of RTT: 42.1% (8/19) hand washing, 26.3% (5/19) bimanual stereotypies associated with each other or with hand mouthing stereotypies, 21% (4/19) hand mouthing, 5.3% (1/19) hand twisting and 5.3% (1/19) hand wringing.

**Figure 1:**
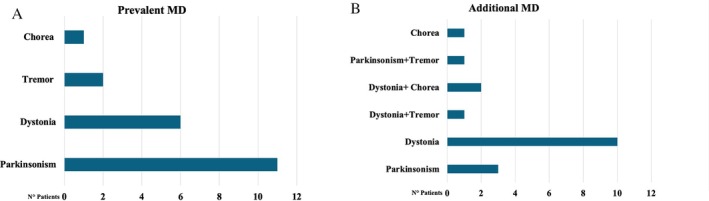
(A) Frequency of prevalent Movement Disorders in patients with Rett Syndrome. (B) Frequency of additional Movement Disorders in patients with Rett Syndrome.

MDs were present in all recruited patients. According to MD‐CRS part I and the neurological/motor assessment, regarding prevalent MD, 55% (11/20) manifested hypokinetic MD (parkinsonism), while 45% (9/20) hyperkinetic (dystonia, tremor, chorea) (Fig. 1A).

Regarding prevalent MD, 11 out of 20 patients (55%) showed parkinsonism (eight were within stage III of the disease, two within stage IV, and one was in stage II). Among the nine patients with prevalent hyperkinetic MD (four in stage II, four in stage III, one in stage IV), 30% (6/20) had dystonia, 10% (2/20) tremor, and 5% (1/20) chorea (Table S2).

Ninety‐Five Percent (19/20) Scored High (>14) on the MD‐CRS Part II Scale, Suggesting a High Impact of MDs on Patients’ Overall Health.

In addition to the prevalent MDs, additional MDs were identified in 90% (18/20) of patients: dystonia in 56% (10/18), parkinsonism in 17% (3/18), chorea in 6% (1/18), and copresence of two disorders in 22% (4/18) dystonia+chorea 11% (2/18), dystonia+tremor 5% (1/18), parkinsonism+tremor 5% (1/18) (Fig. 1B).

Dystonia was present in 95% (19/20), as prevalent MD in six patients and additional (either alone or in combination) in 13. Dystonia severity was graded according to the BFMDRS: 6/19 (32%) patients had a moderate to severe degree of dystonia. Eight out of 19 (42.1%) patients had generalized dystonia, 8/19 (42.1%) multifocal, 2/19 (10.5%) segmental in the neck and trunk, and 1/19 (5.3%) focal cervical dystonia. Twelve out of 19 patients (63.2%) showed persistent dystonia (with worsening during movement in one case), and 7/19 (36.8%) had dystonia with a fluctuating pattern, with exacerbation during activity.

Parkinsonism and Dystonia (Assessed as both Prevalent and Additional MD) Coexisted in 75% (15/20) of Patients (Table S2).

The ICARS subscale of posture and gait revealed high scores in 90% (18/20) of patients. Furthermore, walking subjects (13/20, 65%) had gait characterized by ataxic aspects and freezing (Table S1).

Regarding the clinical aspects assessed, 75% (15/20) of patients had seizures (60% of whom had drug‐resistant epilepsy), 50% (10/20) respiratory disorders, 60% (12/20) gastrointestinal problems, and 40% (8/20) sleep disorders (Table S1).

Statistical analysis revealed that patients without independent ambulation had significantly higher scores on the CSS (*P* < 0.001), and subjects with respiratory and gastrointestinal disorders had a significant association with more severe scores on the BFMDRS (*P* < 0.05; p = 0.02) (Table 1). Furthermore, patients with higher CSS severity had significantly higher scores on MD‐CRS Part I (*P* = 0.03), BFMDRS (*P* = 0.01), and ICARS (*P* = 0.04) (Table S3). Finally, those with high RARS scores had a significant correlation with MD‐CRS Part I and II scores (*P* = 0.03, *P* = 0.003) and the ICARS subscale (*P* = 0.01) (Table S4).

**TABLE 1:** Comparison of the presence or absence of ambulation, respiratory, and gastrointestinal disorders with respect to age at assessment, disease severity (based on CSS and RARS scale scores), manual function (Hand Apraxia), and frequency and severity of movement disorders (based on MD‐CRS, BFMDRS, ICARS scale scores)

	Ambulation (present)	Ambulation (absent)	*P*_value	Breathingdisorder (present)	Breathing disorder (absent)	*P*_value	GI disorder (present)	GI disorder (absent)	*P*_value
	*N*°= 13	*N*°= 7		*N*°= 10	*N*°= 10		*N*°= 12	*N* = 8	
Median (min; max) Median (min; max) Median (min; max)									
Age at evaluation (y)	10 (5;39)	12 (3;40)	0.88	12,50 (8; 40)	6 (3; 31)	**0.003**	11,50 (3; 39)	9 (5; 40)	0.62
CSS (Total score)	23 (13; 34)	30 (26; 44)	**<0.001**	25,5 (15; 44)	25,5 (13; 30)	0.31	24 (13; 44)	26,5 (15; 41)	0.85
RARS (Total score)	74,5 (63,5; 95,5)	83,5 (79,5; 96,5)	0.06	84,25 (63,5; 96,5)	78,75 (66,5; 87)	0.25	79,75 (68,5; 96,5)	82 (63,5; 95,5)	0.97
Hand Apraxia	2 (0; 9)	1 (0; 3)	0.39	1 (0; 8)	1,5 (0; 9)	0.53	0,5 (0; 9)	1,5 (0; 8)	0.21
MD‐CRS (Part I)	36 (26; 51)	50 (33; 55)	0.08	48 (34; 55)	36 (26; 42)	**0.03**	36 (26; 55)	39,5 (34; 52)	0.52
MD‐CRS (Part II)	13 (6; 17)	14 (3; 28)	0.44	14 (6; 28)	11,5 (3; 17)	**0.03**	13,5 (3; 28)	13 (6; 24)	1
BFMDRS (Total score)	30 (0; 77)	35,5 (24; 60)	0.31	41 (4,5; 77)	22,25 (0; 60)	**0.05**	41 (4; 77)	23 (0; 35,5)	**0.02**
ICARS (Total score)	82 (63; 92)	93 (79; 96)	**0.003**	87,5 (78; 94)	83,5 (63; 96)	0.74	86 (63; 96)	83,5 (78; 94)	0.73
ICARS‐ Posture and Gait score	21 (13; 32)	32 (27; 34)	**<0.001**	26 (17; 34)	23 (13; 32)	0.68	24 (13; 34)	25 (17; 34)	0.91

*Note*: Differences between groups were evaluated using the Mann–Whitney *U* test for continuous variables. A *P* value of <0.05 was considered to indicate statistical significance; all *P* values were based on two‐tailed tests.

‐Clinical Severity Scale (CSS): scores >21 indicated greater RTT severity.

‐Rett Assessment Rating Scale (RARS): scores 0–54 mild, 55–80 moderate, and 81–128 severe.

‐Hand Apraxia Scale: scores 0–4: absent/minimal manual function; 5–10: major/maximum level of manual function.

‐Movement Disorders‐Childhood Rating Scale (MD‐CRS): general assessment‐Part I (scores >30 severe MD); MD assessment‐Part II (scores >14 greater severity).

‐Burke‐Fahn‐Marsden Dystonia Rating Scale (BFMDRS): distinguished dystonia types and frequency, severity graded as mild (0–40), moderate (41–80), and severe (81–120).

‐International Cooperative Ataxia Rating Scale (ICARS), posture and gait subscale: scores 0–17 mild ataxia; scores 18–34: severe ataxia.

‐Ambulation was assessed based on clinical and motor examinations.

‐Breathing/GI disorders were evaluated based on clinical examinations and caregivers’ reports.

Abbreviations: BFMDRS, Burke‐Fahn‐Marsden Dystonia Rating Scale; CSS, Clinical Severity Scale; GI, Gastrointestinal; ICARS, International Co‐operative Ataxia Rating Scale; MD‐CRS, Movement disorders‐childhood rating scale; RARS, Rett Assessment Rating Scale.

## Discussion

Our study analyzed 20 female subjects with RTT; all had a prevalent MD: 55% of the sample exhibited parkinsonism (hypokinetic‐type MD), and 45% hyperkinetic‐type MD, including dystonia, chorea, and tremors (Fig. 1A). In 90% of patients, additional MD was present (Fig. 1B).

According to Brunetti et al, in our group, hypokinetic and hyperkinetic MDs appeared in patients regardless of age and stage of the disease (hyperkinetic MDs at 3 years of age, hypokinetic at 5 years).^11^

Dystonia was the most frequent MD, present as either a prevalent or additional (Table S2). In previous studies, the progressive nature of dystonia in RTT was attributed to dysfunction of the dopaminergic system, particularly iron deposition in the basal ganglia.^22^ In our cohort, two of the three oldest patients (31 and 40 years old) did not demonstrate worsening or severe dystonia compared with the younger patients. However, the limited sample size precludes the generation of definitive conclusions.

Tremor and chorea were less common, occurring as prevalent MDs in 10% and 5% of patients, respectively; our results are consistent with previous works.^11^, ^23^

Given the phenotypic complexity of RTT patients, multiple MDs may coexist in the same patient. In particular, it could be complex discerning when rigidity is related to dystonia or parkinsonism since these two MDs share the same brain circuits.^10^, ^24^ Parkinsonism and dystonia coexisted (either as the prevalent or additional disease) in 75% of cases in our sample, highlighting their strong interrelationship (Video 1, Table S2). The pathogenesis of MDs in RTT involves dopaminergic pathways and basal ganglia dysfunction, particularly the reduction of the substantia nigra pars compacta.^24^ Loss‐of‐function variants of *MECP2* could contribute, impairing dopamine synthesis and causing motor abnormalities in RTT.^25^

**Video 1:** 10‐year‐old patient. Hypomimia and mild anterocollis are observed. The upper extremities were flexed with bilateral dystonic posture and choreiform movements at the fingers of the hands. Gait ataxia is associated with freezing, dystonic posture of the lower extremities, and loss of balance.

Independent gait was maintained in 65% of patients and was associated with lower MD‐CRS and BFMDRS scores, reflecting less severe disease progression. Significant associations were found between lack of independent ambulation and higher MD and CSS scores, reinforcing the role of MDs in RTT disease progression. Patients’ walking is often characterized by an ataxic gait and freezing, which could provide new insights into the mechanisms underlying motor dysfunction.

Manual stereotypies, a hallmark of RTT, were present in 95% of the patients, predominantly bimanual, in agreement with a previous study.^10^ Our study did not observe a reduction in the frequency of stereotypies in older patients, supporting findings from Downs et al.^26^

Regarding the clinical characteristics described in RTT, in our cohort, epilepsy (including drug‐resistant forms) was not directly correlated with MD presence or severity. In our sample, 16 patients were taking antiseizure medications (ASMs), 9 of whom were on polytherapy. ASMs may contribute to the development of MDs (mainly dyskinesias and tremor), particularly in females.^27^ However, we observed that off‐treatment patients presented moderate/severe MDs as well, and, on the contrary, the only patient with lower MD scale scores was on ASM bi‐therapy. The small sample size limited the generalizability of these findings.

A significant association was found between dystonia and both respiratory and gastrointestinal problems (Table 1). To our knowledge, the literature has not focused on the co‐occurrence of respiratory disorders and dystonia in RTT. Severe dystonia affecting the trunk could impair respiratory mechanics, increasing the risk of scoliosis and breathing difficulties. Similarly, we hypothesized that the association between dystonia and gastrointestinal problems may be due to dyskinetic and dystonic oral movements during feeding: fluoroscopic studies have highlighted the detrimental impact of oral MDs on gastrointestinal function.^28^ The mechanisms underlying this association are poorly understood and need further study.

The small sample size and the focus on clinically complex patients at a tertiary pediatric center have precluded the generalizability of the results. Most patients were found to have parkinsonism as the MD prevalent; however, the Unified Parkinson's Disease Rating Scale was not used due to severe cognitive impairment preventing task performance, resulting in high scores and poor discrimination.^29^

MDs should be systematically evaluated (with standardized tests and video‐recording of the neurological examination) in RTT patients, potentially influencing clinical guidelines and personalized treatment strategies.

## Author Roles

(1) Research project: A. Conception, B. Organization, C. Execution; (2) Statistical Analysis: A. Design, B. Execution, C. Review and Critique; (3) Manuscript Preparation: A. Writing of the first draft, B. Review and Critique.

S.B.: 1B, 1C, 3A

G.P.: 1A, 1B

M.G.C.: 2A, 2B, 2C

S.C.: 1C

L.N.: 3B

E.D.G.: 1A, 1B, 75(12):369‐393.3B

## Disclosures

**Ethical Compliance Statement:** The study was conducted according to the 1964 Declaration of Helsinki. Parents provided signed informed consent to video‐record the neurological objective exam. We confirm that we have read the Journal's position on issues involved in ethical publication and affirm that this work is consistent with those guidelines.

**Funding Sources and Conflict of Interest:** No specific funding was received for this work. The authors declare that there are no conflicts of interest relevant to this work.

**Financial Disclosures for the Previous 12 Months:** The authors declare that there are no additional disclosures to report.

## Supporting information


**Supplementary Table S1.** Clinical and MD characteristics of patients.

**Supplementary Table S2.** Scores of disease and MD rating scales and classification of prevalent and additional MD for each patient.

**Supplementary Table S3.** Comparison of disease severity (based on CSS and RARS scale scores) with respect to age at assessment, manual function (Hand Apraxia), and severity of movement disorders (based on MD‐CRS, BFMDRS, ICARS scale scores).

**Supplementary Table S4.** Spearman correlation between scores of RARS scale and movement disorders scales.

## Data Availability

The data that supports the findings of this study are available in the supplementary material of this article.
