# Supplementary material for: Parkinsonism and Dystonia Are Prevalent and Concomitant Movement Disorders in a Cohort of Patients with Rett Syndrome
Source: Mov Disord Clin Pract. 2025 May 30;12(11):1843–8. doi: 10.1002/mdc3.70158 (PMC12995117; doi:10.1002/mdc3.70158)
Supplement: Supplementary file 2 — Supplementary Table S1. Clinical and MD characteristics of patients. [file MDC3-12-1843-s001.docx]

**Supplementary Table 1** Clinical and MD characteristics of patients

|  | N= 20 |
| --- | --- |
| Median age at evaluation | 11 y (range 3-40) |
| CSS (severe) | 17 (85%) |
| RARS (severe) | 10 (50%) |
| Hand Apraxia (severe) | 15 (75%) |
| Hand Stereotypies | 19 (95%) |
| MD Hypokinetic (prevalent) | 11 (55%) |
| MD Hyperkinetic (prevalent) | 9 (45%) |
| MD-CRS part II (severe) | 5 (25%) |
| BFMDRS (moderate/severe) | 6 (30%) |
| ICARS (severe) | 18 (90%) |
| Ambulation (present) | 13 (65%) |
| Epilepsy (present) | 15 (75%) |
| Drug-resistant Epilepsy | 9 (45%) |
| Sleep disorders (present) | 9 (45%) |
| Breathing disorders (present) | 10 (50%) |
| Gastrointestinal disorders (present) | 12 (60%) |
| Cardiovascular disorders (present) | 0 |

**Abbreviations**: N: number of patients; CSS: Clinical Severity Scale, RARS: Rett Assessment Rating Scale, MD: movement disorder, MD-CRS: Movement Disorders-Childhood Rating Scale, BFMDRS: Burke-Fahn-Marsden Dystonia Rating Scale, ICARS: International Cooperative Ataxia Rating Scale.

**Footnote**:

-Clinical Severity Scale (CSS): scores >21 indicated greater RTT severity

-Rett Assessment Rating Scale (RARS): scores 0-54 mild, 55-80 moderate, and 81-128 severe

-Hand Apraxia Scale: scores 0-4: absent/minimal manual function; 5- 10: major/maximum level of manual function

-Movement Disorders-Childhood Rating Scale (MD-CRS): general assessment-Part I (scores > 30 severe MD); MD assessment-Part II (scores > 14 greater severity).

-Burke-Fahn-Marsden Dystonia Rating Scale (BFMDRS): distinguished dystonia types and frequency, severity graded as mild (0-40), moderate (41-80), and severe (81-120)

-International Cooperative Ataxia Rating Scale (ICARS), posture and gait subscale: scores 0-17 mild ataxia; scores 18-34: severe ataxia

-Ambulation was assessed based on clinical and motor examinations

-Sleep/Breathing/GI/Cardiovascular disorders were evaluated based on clinical examinations and caregivers’ reports.

-Epilepsy and drug- resistant epilepsy were evaluated based on caregivers’ reports and EEG tracing.
