# Supplementary material for: Parkinsonism and Dystonia Are Prevalent and Concomitant Movement Disorders in a Cohort of Patients with Rett Syndrome
Source: Mov Disord Clin Pract. 2025 May 30;12(11):1843–8. doi: 10.1002/mdc3.70158 (PMC12995117; doi:10.1002/mdc3.70158)
Supplement: Supplementary file 3 — Supplementary Table S2. Scores of disease and MD rating scales and classification of prevalent and additional MD for each patient. [file MDC3-12-1843-s004.docx]

**Supplementary Table 2** Scores of disease and MD rating scales and classification of prevalent and additional MD for each patient

| ID | Age at evaluation | MECP2 variant | CSS | RARS | Hand Apraxia | Prevalent MD | Additional MD | MD-CRS Part I | MD-CRS Part II | BFMDRS | Dystonia type | ICARS |
| --- | --- | --- | --- | --- | --- | --- | --- | --- | --- | --- | --- | --- |
| **GE_01** | 39 | c.372G>C (p.Leu124Phe) | 21 | 74.5 | 0 | Dystonia | Parkinsonism | 51 | 14 | 77 | Generalized | 78 |
| **GE_02** | 4 | c.1203_1231del (p.Pro402Hisfs*5) | 30 | 79.5 | 3 | Dystonia | Absent | 36 | 3 | 24 | Multifocal | 90 |
| **GE_03** | 40 | c.916C>T (p.Arg306Cys) | 27 | 83 | 1 | Parkinsonism | Dystonia+Tremor | 51 | 14 | 28 | Segmental (Neck-Trunk) | 92 |
| **GE_04** | 13 | c.401C>T (p.Ser134Phe) | 23 | 68.5 | 4 | Dystonia | Parkinsonism +tremor | 34 | 13 | 63 | Generalized | 81 |
| **GE_05** | 12 | c.880C>T (p.Arg 294*) | 15 | 63.5 | 8 | Parkinsonism | Dystonia (mild) | 34 | 6 | 4.5 | Multifocal | 78 |
| **GE_06** | 9 | c.502C>T (p.Arg168*) | 34 | 90 | 1 | Parkinsonism | Dystonia+chorea | 46 | 14 | 54.5 | Generalized | 88 |
| **GE_07** | 8 | c.808 C>T (p.Arg270*) | 22 | 74 | 0 | Parkinsonism | Dystonia | 42 | 11 | 34.5 | Focal | 81 |
| **GE_08** | 5 | c.952C>T (p.Arg318Cys) | 26 | 74.5 | 5 | Chorea | Absent | 37 | 12 | 0 | Absent | 92 |
| **GE_09** | 12 | c.808 C>T (p.Arg270*) | 38 | 85.5 | 0 | Parkinsonism | Dystonia | 50 | 28 | 44 | Multifocal | 93 |
| **GE_10** | 11 | c.401C>G (p.Ser134Cys) | 13 | 74.5 | 9 | Parkinsonism | Dystonia (mild) | 26 | 14 | 4 | Multifocal | 63 |
| **GE_11** | 12 | c.502C>T (p.Arg168*) | 41 | 90.5 | 1 | Parkinsonism | Dystonia | 52 | 24 | 29.5 | Segmental (Neck-Trunk) | 94 |
| **GE_12** | 8 | c.1157_1186 delinsA | 24 | 95.5 | 0 | Dystonia | Parkinsonism | 38 | 16 | 30 | Generalized | 78 |
| **GE_13** | 14 | c.502C>T (p.Arg168*) | 44 | 96.5 | 0 | Dystonia | Parkinsonism | 55 | 23 | 58 | Generalized | 94 |
| **GE_14** | 5 | c.502C>T (p.Arg168*) | 21 | 84 | 0 | Parkinsonism | Dystonia | 33 | 8 | 20.5 | Multifocal | 82 |
| **GE_15** | 10 | c.473C>T (p.Thr158Met) | 27 | 87 | 2 | Parkinsonism | Dystonia+chorea | 41 | 17 | 18 | Generalized (mild) | 85 |
| **GE_16** | 5 | c.401C>G (p.Ser134Cys) | 15 | 66.5 | 8 | Tremor | Dystonia | 34 | 9 | 17 | Multifocal | 82 |
| **GE_17** | 7 | c.316C>T (p.Arg106Trp) | 28 | 81 | 1 | Dystonia | Chorea | 41 | 11 | 35.5 | Multifocal | 79 |
| **GE_18** | 21 | c. 1194-6968 deletion of exon 4 | 23 | 80 | 5 | Parkinsonism | Dystonia | 35 | 13 | 38 | Generalized | 87 |
| **GE_19** | 3 | c.538C>T, (p.Arg180*) | 26 | 83.5 | 1 | Tremor | Dystonia | 33 | 12 | 60 | Multifocal | 96 |
| **GE_20** | 31 | c.916C>T (p.Arg306Cys) | 25 | 78 | 0 | Parkinsonism | Dystonia | 36 | 14 | 35 | Generalized | 85 |

**Abbreviations**: CSS: Clinical Severity Scale, RARS: Rett Assessment Rating Scale, MD: movement disorder, MD-CRS: Movement Disorders-Childhood Rating Scale, BFMDRS: Burke-Fahn-Marsden Dystonia Rating Scale, ICARS: International Cooperative Ataxia Rating Scale.

**Footnote**:

-Clinical Severity Scale (CSS): scores >21 indicated greater RTT severity

-Rett Assessment Rating Scale (RARS): scores 0-54 mild, 55-80 moderate, and 81-128 severe

-Hand Apraxia Scale: scores 0-4: absent/minimal manual function; 5- 10: major/maximum level of manual function

-Movement Disorders-Childhood Rating Scale (MD-CRS): general assessment-Part I (scores > 30 severe MD); MD assessment-Part II (scores > 14 greater severity).

-Burke-Fahn-Marsden Dystonia Rating Scale (BFMDRS): distinguished dystonia types and frequency, severity graded as mild (0-40), moderate (41-80), and severe (81-120)

-International Cooperative Ataxia Rating Scale (ICARS), posture and gait subscale: scores 0-17 mild ataxia; scores 18-34: severe ataxia
