# Supplementary material for: Parkinsonism and Dystonia Are Prevalent and Concomitant Movement Disorders in a Cohort of Patients with Rett Syndrome
Source: Mov Disord Clin Pract. 2025 May 30;12(11):1843–8. doi: 10.1002/mdc3.70158 (PMC12995117; doi:10.1002/mdc3.70158)
Supplement: Supplementary file 4 — Supplementary Table S3. Comparison of disease severity (based on CSS and RARS scale scores) with respect to age at assessment, manual function (Hand Apraxia), and severity of movement disorders (based on MD‐CRS, BFMDRS, ICARS scale scores). [file MDC3-12-1843-s002.docx]

**Supplementary Table 3** Comparison of disease severity (based on CSS and RARS scale scores) with respect to age at assessment, manual function (Hand Apraxia), and severity of movement disorders (based on MD-CRS, BFMDRS, ICARS scale scores).

|  | **CSS (mild) N°=3** | **CSS (severe)**  **N°=17** | **P_value** | **RARS (mild)**  **N°=10** | **RARS (severe)**  **N°=10** | **P_value** |
| --- | --- | --- | --- | --- | --- | --- |
| **Median (min; max) Median (min; max)** | | | | | | |
| **Age at evaluation (y)** | 11 (5; 12) | 10 (3; 40) | 0.76 | 11,5 (4; 39) | 9,5 (3; 40) | 0.68 |
| **Hand Apraxia** | 8 (8; 9) | 1 (0; 5) | **0.002** | 4,5 (0; 9) | 1 (0; 2) | **0.05** |
| **MD-CRS (Part I)** | 34 (26; 34) | 41 (33; 55) | **0.03** | 35,5 (26; 51) | 43,5 (33; 55) | 0.07 |
| **MD-CRS (Part II)** | 9 (6; 14) | 14 (3; 28) | 0.22 | 12,5 (3; 14) | 15 (8; 28) | **0.04** |
| **BFMDRS (Total score)** | 4,5 (4; 17) | 35 (0; 77) | **0.01** | 29,25 (0; 77) | 32,75 (18; 60) | 0.39 |
| **ICARS (Total score)** | 78 (63; 82) | 87 (78; 96) | **0.04** | 81,5 (63; 92) | 90 (78; 96) | 0.06 |
| **ICARS- Posture and Gait (score)** | 17 (13; 21) | 27 (18; 34) | **0.02** | 21 (13; 32) | 29,5 (18; 34) | **0.03** |

**Abbreviation:** CSS: Clinical Severity Scale; RARS: Rett Assessment Rating Scale; MD-CRS: Movement disorders-childhood rating scale; BFMDRS: Burke-Fahn-Marsden Dystonia Rating Scale; ICARS: International Co-operative Ataxia Rating Scale; GI: Gastrointestinal

**Footnote**: Differences between groups were evaluated using the Mann-Whitney U test for continuous variables. A P value of <0.05 was considered to indicate statistical significance; all P values were based on two-tailed tests.

-Clinical Severity Scale (CSS): scores >21 indicated greater RTT severity

-Rett Assessment Rating Scale (RARS): scores 0-54 mild, 55-80 moderate, and 81-128 severe

-Hand Apraxia Scale: scores 0-4: absent/minimal manual function; 5- 10: major/maximum level of manual function

-Movement Disorders-Childhood Rating Scale (MD-CRS): general assessment-Part I (scores > 30 severe MD); MD assessment-Part II (scores > 14 greater severity).

-Burke-Fahn-Marsden Dystonia Rating Scale (BFMDRS): distinguished dystonia types and frequency, severity graded as mild (0-40), moderate (41-80), and severe (81-120)

-International Cooperative Ataxia Rating Scale (ICARS), posture and gait subscale: scores 0-17 mild ataxia; scores 18-34: severe ataxia
