# Supplementary material for: Parkinsonism and Dystonia Are Prevalent and Concomitant Movement Disorders in a Cohort of Patients with Rett Syndrome
Source: Mov Disord Clin Pract. 2025 May 30;12(11):1843–8. doi: 10.1002/mdc3.70158 (PMC12995117; doi:10.1002/mdc3.70158)
Supplement: Supplementary file 5 — Supplementary Table S4. Spearman correlation between scores of RARS scale and movement disorders scales. [file MDC3-12-1843-s003.docx]

**Supplementary Table 4** Spearman correlation between scores of RARS scale and movement disorders scales

|  | **RARS (Total score)** | |
| --- | --- | --- |
|  | Correlation coefficient | P_value |
| **MD- CRS (Part I)** | 0,495 | **0,03** |
| **MD- CRS (Part II)** | 0,637 | **0,003** |
| **BFMDRS (Total score)** | 0,256 | 0,28 |
| **ICARS (Total score)** | 0,501 | **0,02** |
| **ICARS- Posture and Gait (score)** | 0,547 | **0,01** |

**Abbreviation:** RARS: Rett Assessment Rating Scale; MD-CRS: Movement disorders-childhood rating scale; BFMDRS: Burke-Fahn-Marsden Dystonia Rating Scale; ICARS: International Co-operative Ataxia Rating Scale

**Footnote**: A P value of <0.05 was considered to indicate statistical significance; all P values were based on two-tailed tests.

-Rett Assessment Rating Scale (RARS): scores 0-54 mild, 55-80 moderate, and 81-128 severe

-Hand Apraxia Scale: scores 0-4: absent/minimal manual function; 5- 10: major/maximum level of manual function

-Movement Disorders-Childhood Rating Scale (MD-CRS): general assessment-Part I (scores > 30 severe MD); MD assessment-Part II (scores > 14 greater severity).

-Burke-Fahn-Marsden Dystonia Rating Scale (BFMDRS): distinguished dystonia types and frequency, severity graded as mild (0-40), moderate (41-80), and severe (81-120)

-International Cooperative Ataxia Rating Scale (ICARS), posture and gait subscale: scores 0-17 mild ataxia; scores 18-34: severe ataxia
